# Comparison of electrocardiogram parameters and echocardiographic response between distinct left bundle branch area pacing modes in heart failure patients

**DOI:** 10.3389/fcvm.2024.1441241

**Published:** 2024-08-29

**Authors:** Yao Li, Wei Zhang, Keping Chen, Zhexun Lian

**Affiliations:** ^1^ Department of Cardiology, The Affiliated Hospital of Qingdao University, Qingdao, China; ^2^ State Key Laboratory of Cardiovascular Disease, Arrhythmia Center, Fuwai Hospital, National Center for Cardiovascular Diseases, Chinese Academy of Medical Sciences and Peking Union Medical College, Beijing, China; ^3^ Department of Urology, The Affiliated Hospital of Qingdao University, Qingdao, China

**Keywords:** left bundle branch area pacing, branch pacing, electrocardiogram, echocardiographic response, heart failure

## Abstract

**Background:**

Left bundle branch area pacing (LBBAP) has become an alternative method for cardiac resynchronization therapy. Various modes of LBBAP have been determined, including left bundle trunk pacing (LBTP), left anterior branch pacing (LAFP) and left posterior branch pacing (LPFP). However, whether the outcomes of various pacing modes differ in heart failure (HF) patients is still unclear. This study aimed to compare the electrophysiological characteristics and echocardiographic response rate among those distinct modes of LBBAP.

**Methods:**

HF patients undergoing successful LBBAP were retrospectively included. Distinct modes of pacing were determined based on paced QRS morphology. The fluoroscopic images were collected to compare the lead tip position between the groups. The electrocardiograms (ECG) before and after LBBAP were used to measure the depolarization (QRS duration [QRSd] and the interventricular delay [IVD]), and the repolarization parameters [QTc, TpeakTend(TpTe), and TpTe/QTc]. The left ventricular ejection fraction (LVEF) and left ventricular end-diastolic diameter (LVEDD) of patients were also recorded. In addition, the lead parameters and certain complications were compared.

**Results:**

A total of 64 HF patients were finally included, consisting of 16 (25.0%) patients in the LBTP group, 22 (34.4%) patients in the LAFP group, and 26 (40.6%) patients in the LPFP group. The distribution features of LBBAP lead tips were significantly related to pacing modes: LBTP was more likely to be in zone 4 while LAFP or LPFP was prone to locate in zone 5. After LBBAP, the ventricular ECG parameters were significantly improved, regardless of pacing modes. Besides, the LVEF of the patients was significantly increased (*P *< 0.001), and LVEDD was significantly decreased (*P *< 0.001). There was no difference in the response rate and super-response rate among groups (*P *> 0.05). In addition, the lead parameters remained stable and no significant difference was observed among groups.

**Conclusion:**

LPFP was the main pacing mode among HF patients after LBBAP. The paced QRS morphology was significantly related to the position of lead tips. After LBBAP, the ventricular depolarization synchronization and repolarization stability were both significantly improved, regardless of pacing modes. There was no significant difference in the echocardiographic response rate among distinct LBBAP modes.

## 1 Introduction

Left bundle branch area pacing (LBBAP), a novel technique of physiological pacing, has become a new option for heart failure (HF) patients indicated for cardiac resynchronization therapy (CRT) (1–3). The safety and viability of this novel technique in HF patients were demonstrated by several studies (2, 4–6). For example, a multi-center, retrospective study involving 325 HF patients found that QRS duration (QRSd) narrowed from 152 ± 32 ms to 137 ± 22 ms and left ventricular ejection fraction (LVEF) improved from 33 ± 10% to 44 ± 11% (*P *< 0.01) after LBBAP (5). Besides, LBBAP was no worse than traditional biventricular pacing (BiVP) concerning reversal of cardiac function in HF patients (7). Even more, the attempt of LBBAP as a rescue strategy among BiVP non-responders or those with failed coronary sinus leads has also achieved prominent effects (8). So far, LBBAP is becoming a universally-accepted way of CRT delivery.

Despite its remarkable performance, LBBAP can be quite a challenge in HF patients with severe electro-mechanical remodeling (9), and the successful rate was reported to be significantly lower than bradycardia patients (82.2 vs. 92.4%) (2). Initially, LBBAP highlighted capture of left bundle branch (LBB) trunk, with location of lead tip 1.5–2 cm distal to His bundle on right ventricle (RV) septum (1, 10, 11). However, the anatomy of LBB trunk varies from patient to patient, and LBB even promptly bifurcates into left anterior fascicle (LAF) and left posterior fascicle (LPF) without the trunk part in some patients (12). Thus, the traditional left bundle trunk pacing (LBTP) methodology resulted in low successful rate, severe septal injury and long radiation time due to repeated attempts. Some experts suggested a wider range of LBBAP targets on septum besides LBB trunk, and various pacing modes with distinct electrocardiogram features thus emerge, such as LBTP, LAF pacing (LAFP) and LPF pacing (LPFP) (2, 13, 14). Such refinement of LBBAP methodology remarkably facilitates this novel technique and promote its extensive application around the world.

However, whether the outcomes differ among those distinct pacing modes in HF patients remains vague. Studies involving bradycardia patients drew controversial conclusions: Lin et al. (15) found LBTP, LAFP and LPFP all obtained similar depolarization synchrony, while Liu et al. (16) reported that LBTP achieved better ventricular synchrony than LAFP or LPFP. So far, the outcomes of various pacing modes in HF patients have never been reported yet. This study aimed to include HF patients undergoing successful LBBAP, and divided them in terms of distinct pacing modes (LBTP, LAFP or LPFP). The relationship between pacing modes and tip location under fluoroscopy was then examined. Besides, ventricular electrogram (ECG) features (depolarization and repolarization parameters) and echocardiographic response after LBBAP were also compared between groups, in order to determine the optimal pacing modality in HF patients.

## 2 Methods

### 2.1 Study population

This was a retrospective study. HF patients who underwent successful LBBAP at Fuwai Hospital from December 2017 to November 2022 were consecutively included. The inclusion criteria were as follows: (1) severe HF symptoms (NYHA class II–IV) despite optimal medication therapy for at least 3 months (17); (2) LVEF < 50%; (3) intrinsic QRSd ≥ 130 ms. All included patients agree on LBBAP operation and the use of relevant clinical data. This study has been approved by the Hospital Ethics Review Committee.

### 2.2 LBBAP procedure and post-operation programming

During the LBBAP operation, the tip of Select-Secure pacing lead (model 3830, 69 cm, Medtronic, Minnesota, USA) was screwed perpendicularly into the interventricular septum through C315 HIS sheath (Medtronic, Minnesota, USA) to reach the LBB area under RAO 30° fluoroscopy (15, 18, 19). The QRS morphology, pacing impedance and stimulus to left ventricular activation time (stim-LVAT) of unipolar pacing should be closely monitored during the rotation. The criteria for successful LBBAP were as follows (5, 20). The QRS morphology of unipolar pacing shows RBBB pattern or correction of LBBB, with at least one of the following items being met: (1) transition from non-selective LBBP to selective LBBP during the process of output reduction at the same pacing site; (2) transition from non-selective LBBP to LVSP during the above mentioned output reduction (abrupt extension of stim-LVAT ≥ 10 ms); (3) stim-LVAT remained short and constant (≤90 ms) despite output changes. All CRT patients undergoing successful LBBAP were programmed with the mode of LBBAP-only and the optimal AV delay was set in pursuit of the narrowest paced QRSd (4, 21).

### 2.3 ECG criteria to determine the pacing modes of LBBAP

**Figure 1:**
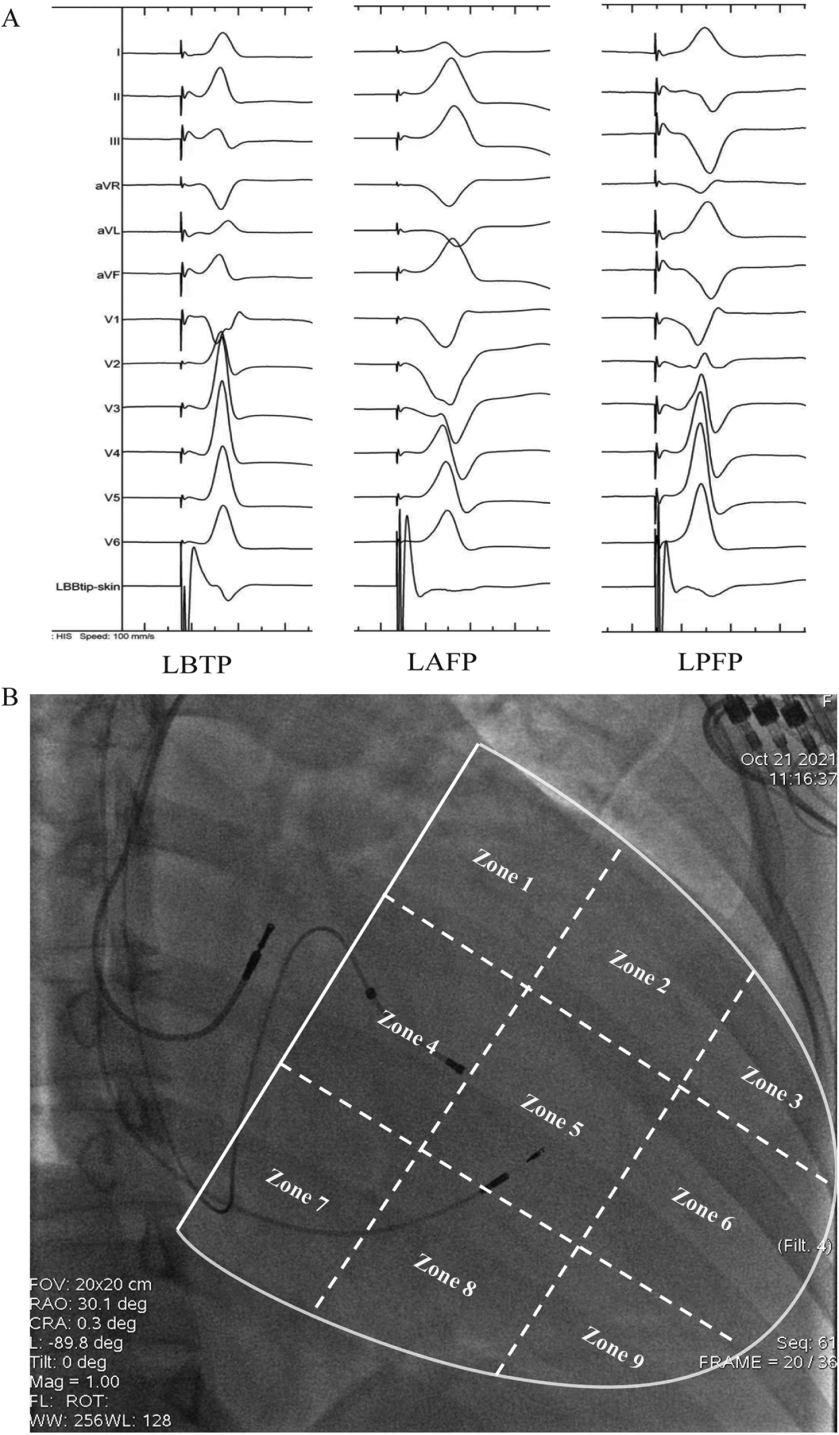
Schematic diagram of grouping based on paced QRS morphology **(A)** and distribution of lead tip using the 9-partition method **(B)**. LAFP, left anterior fascicle pacing; LBTP, left bundle trunk pacing; LPFP, left posterior fascicle pacing.

The QRS morphology of LBBAP under VVI mode and unipolar pacing was analyzed to determine the paced branch of left bundle (LBTP, LAFP or LPFP). The paced ECG criteria were as follows (2, 15, 16): (1) LBTP: frontal QRS axis close to normal; dominantly positive QRS waves in lead II; negative component present in lead III; (2) LAFP: right-axis deviation; dominantly positive QRS waves in lead II and lead III; dominantly negative waves in leads I and aVL; (3) LPFP: left-axis deviation; dominantly negative QRS waves in lead II and lead III; dominantly positive waves in leads I and aVL (Figure 1A).

### 2.4 Fluoroscopic location of the LBBAP lead tip with the 9-partition method

As previously described, ventricle boundary between atrioventricular groove and apex was divided into 9 partitions under RAO 30° fluoroscopy (22). Briefly, a line through coronary sinus ostium (a transparent area under fluoroscopy) and meanwhile parallel to atrioventricular junction was identified as the atrioventricular groove, which was then divided into three identical parts by two vertical lines along the long-axis of ventricle. The long-axis lines were then divided into three identical parts by two short-axis lines parallel to atrioventricular groove. With these four lines, the area between atrioventricular groove and ventricular apex was divided into 3 × 3 partitions. The partition diagram is shown in Figure 1B.

### 2.5 Measurement of ECG parameters

**Figure 2:**
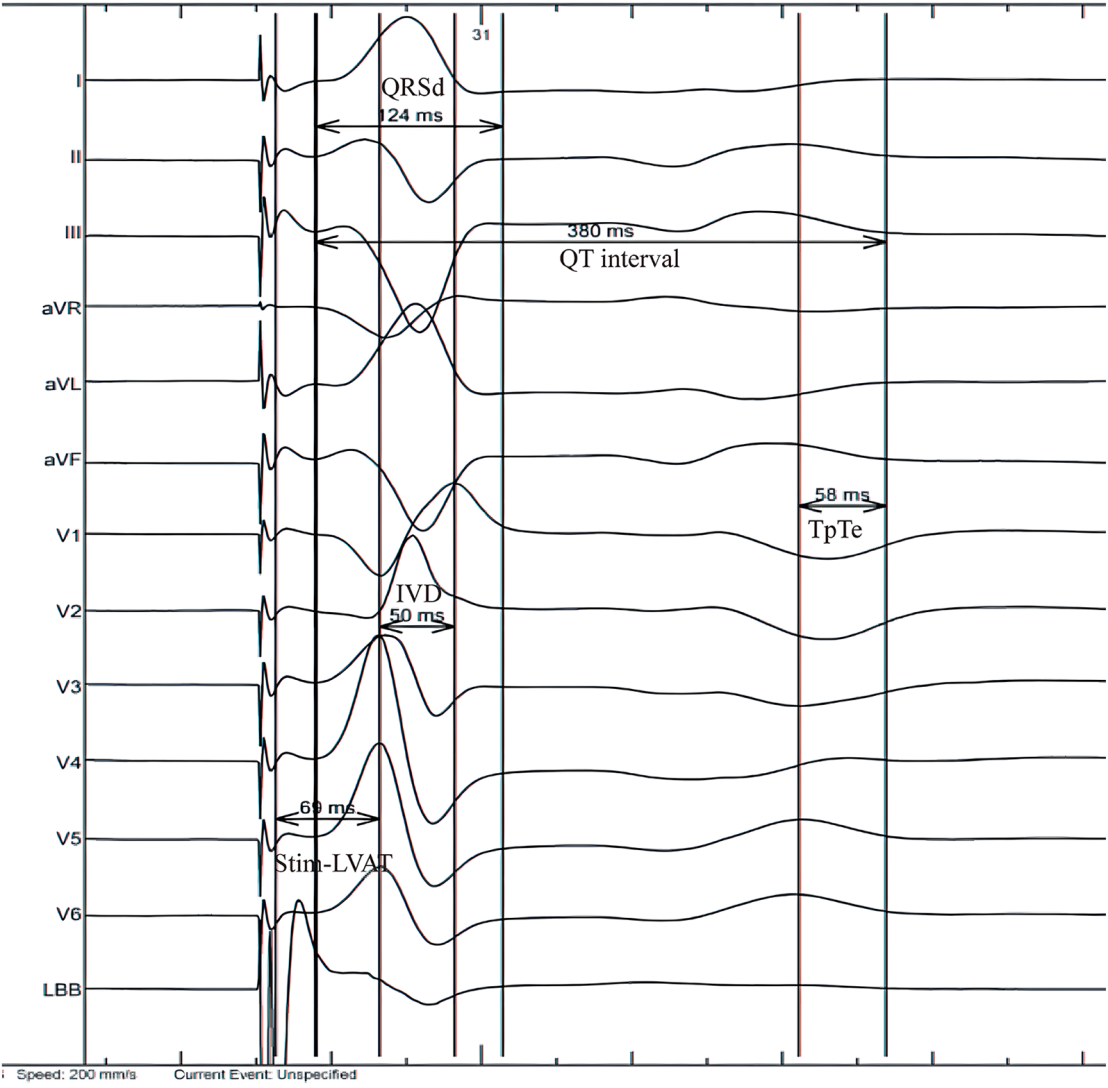
Measurement of electrocardiogram parameters. IVD, interventricular delay; QRSd, QRS duration; Stim-LVAT, stimulus to left ventricular activation time; TpTe, TpeakTend.

In this study, the ECGs under intrinsic rhythm before operation and the paced ECGs (LBBAP unipolar pacing, at 5 v/0.4 ms, VVI mode) 24 h after operation were included. The RR interval, QRSd, stim-LVAT, interventricular delay (IVD), QT interval and TpTe interval were all measured. QRSd is measured from the earliest onset to the latest end of QRS waves among all 12 leads. Stim-LVAT is the time interval from the beginning of the stimulus artifacts to the peak of R wave in lead V5 or V6. The IVD interval is the time interval between the peak of R wave in lead V1 and lead V6 (if the R wave in lead V6 has notch, the distance should be measured to the second peak). Repolarization parameters were measured in lead V5 (if the T wave in V5 is flat or vague, using V4 or V6 instead). QT interval is the distance between the beginning of QRS and the end of T wave. The end of T wave was defined as the intersection point of the steepest tangent of the descending branch of T wave and the equipotential line (23). QTc is the QT interval corrected by Bazett formula according to heart rate (24). TpTe is the distance from the peak of T wave to the end of T wave and it is measured from the valley of T wave if the T wave is negative or negative (25) (Figure 2). All parameters were measured for 5 consecutive heat beats and then the averages were finally taken (26). Of note, TpTe is the main repolarization indicators in this study, while QTc and TpTe/QTc are the secondary indicators, since the former parameter reflected repolarization more accurately than other repolarization indicators (27).

### 2.6 Echocardiogram

All CRT patients underwent transthoracic two-dimensional echocardiography 1 week before and at least 3 months after the implantation at our center. The baseline and follow-up LVEF and LVEDD from the medical record system were collected. LVEF is measured using the biplane Simpson method. Positive echocardiographic response was defined as an increase of LVEF ≥ 5% at follow-up (5). If LVEF increased by at least 20% compared to the baseline and/or increased to more than 50% in those with a baseline LVEF ≤ 35%, the patient is considered a super-responder (28).

### 2.7 Statistics

Continuous variables accorded with normal distribution were represented by mean ± standard deviation, independent or paired sample *t*-tests were used for comparison between two groups, and one-way ANOVA was used for comparison between three groups (LSD for *post hoc* multiple comparisons). Those continuous variables with skew distribution were represented by the median (interquartile range), the Mann Whitney *U*-test or Wilcoxon signed rank test was used for comparison between two groups, and the Kruskal Wallis test was used for comparison between the three groups. The categorical variables were represented by numbers and percentages, group comparisons were performed using chi square tests or Fisher's exact tests, and pairwise comparisons between multiple groups are corrected using the Bonferroni method. All statistical tests were bilateral, and *P* < 0.05 was considered statistically significant.

## 3 Results

### 3.1 Basic characteristics of the study population

Among the 75 patients who ever tried LBBAP, a total of 64 HF patients were finally included in the study with a success rate of 85.3%, including 16 (25.0%) in the LBTP group, 22 (34.4%) in the LAFP group, and 26 (40.6%) in the LPFP group (Table 1). 60.9% of the patients in the study population were male, and 71.9% had baseline LBBB [Strauss criteria (29, 30)]. The total population had an average QRSd of 181.44 ± 17.13 ms and an average LVEF value of 32.72 ± 6.91%. Baseline features were relatively comparable among the three groups (Table 1).

**Table 1:** Basic characteristics of the study population.

Variables	All patients (*n* = 64)	LBTP (*n* = 16)	LAFP (*n* = 22)	LPFP (*n* = 26)	*P* value
Gender (%, male)	39 (60.9)	10 (62.5)	12 (54.5)	17 (65.4)	0.74
Age (year)	61.31 ± 11.78	58.75 ± 13.21	67.63 ± 8.33	62.91 ± 8.26	0.48
Hypertension (%)	31 (48.4)	6 (37.5)	13 (59.1)	12 (46.2)	0.40
Diabetes (%)	11 (17.2)	4 (25.0)	2 (9.1)	5 (19.2)	0.39
Af (%)	9 (14.1)	3 (18.8)	4 (18.2)	2 (7.7)	0.46
CAD (%)	24 (37.5)	8 (50.0)	7 (31.8)	9 (34.6)	0.48
ICM (%)	12 (18.8)	3 (18.8)	5 (22.7)	4 (15.4)	0.81
Hyperlipidemia (%)	35 (54.7)	8 (50.0)	13 (59.1)	14 (53.8)	0.85
CKD (%)	6 (9.4)	1 (6.3)	4 (18.2)	1 (3.8)	0.22
LBBB (%)	46 (71.9)	12 (75.0)	16 (72.7)	18 (69.2)	0.94
QRSd (ms)	181.44 ± 17.13	184.87 ± 16.59	180.18 ± 16.52	180.39 ± 18.30	0.66
LVEF (%)	32.72 ± 6.91	31.75 ± 7.09	31.31 ± 5.42	33.36 ± 7.83	0.55
LVEDD (mm)	65.82 ± 8.82	65.50 ± 8.46	65.31 ± 8.10	68.18 ± 10.93	0.70
*β*-receptor blocker (%)	63 (98.4)	15 (93.8)	22 (100.0)	26 (100.0)	0.15
ACEI/ARB (%)	53 (82.8)	12 (75.0)	17 (77.3)	24 (92.3)	0.22
Amiodarone(%)	20 (31.3)	8 (50.0)	6 (27.3)	6 (23.1)	0.17

ACEI/ARB, angiotensin converting enzyme inhibitor/angiotensin receptor antagonist; Af, atrial fibrillation; CAD, Coronary Artery Disease; CKD, chronic kidney disease; ICM, ischemic cardiomyopathy; LAFP, left anterior fascicle pacing; LBBB, left bundle branch block; LBTP, left bundle trunk pacing; LPFP, left posterior fascicle pacing; LVEDD, left ventricular end diastolic diameter; LVEF, left ventricular ejection fraction; QRSd, QRS duration.

### 3.2 Pacing parameters and complications

The parameters of the LBBAP lead during the procedure were acceptable, with pacing threshold, R-wave amplitude, pacing impedance, and stim-LVAT values of 0.5 (0.5, 0.5) V/0.4 ms, 9.85 ± 4.96 mV, 599.73 ± 139.10 Ω, and 74.24 ± 10.39 ms, respectively. After an average follow-up of 6.75 ± 4.12 months, the R-wave amplitude remained stable (11.78 ± 4.82 mV, *P *> 0.05), the threshold slightly increased [0.5 (0.5, 0.8) V/0.4 ms, *P *< 0.01], and the impedance slightly decreased (464.36 ± 83.66 Ω, *P *< 0.01). Of note, no significant difference was observed among LBTP group, LAFP group, and LPFP group for both baseline and follow-up parameters (Table 2). Only one patient in the study population experienced RBBB, and did not recover after 15 months of follow-up (LPFP, Zone 5), and no other operation-related complications were observed.

**Table 2:** Comparison of pacing parameters between different pacing modes of LBBAP (*n* = 64).

Parameters	LBTP (*n* = 16)	LAFP (*n* = 22)	LPFP (*n* = 26)	*P* value
stim-LVAT (ms)	86.00 ± 5.66	74.00 ± 9.66	71.00 ± 8.37	0.39
Post-operative R wave (mV)	14.35 ± 1.48	9.46 ± 4.25	11.13 ± 6.74	0.86
Post-operative impedance (Ω)	572.50 ± 238.30	499.43 ± 82.61	570.83 ± 95.02	0.054
Post-operative threshold (V/0.4 ms)	0.5 (0.5, 0.5)	0.5 (0.5, 0.5)	0.5 (0.5, 0.5)	0.99
Follow-up R wave (mV)	15.35 ± 6.58	12.56 ± 5.50	9.68 ± 2.99	0.32
Follow-up impedance (Ω)	538.00 ± 45.25	466.86 ± 85.58	482.50 ± 102.44	0.22
Follow-up threshold (V/0.4 ms)	0.5 (0.5, 0.8)	0.5 (0.5, 0.8)	0.5 (0.5, 0.8)	0.73
Follow-up time (month)	10.50 ± 2.12	6.00 ± 4.30	7.30 ± 4.93	0.38

LAFP, left anterior fascicle pacing; LBBAP, left bundle branch area pacing; LBTP, left bundle trunk pacing; LPFP, left posterior fascicle pacing; stim-LVAT, stimulus to left ventricle activation time.

### 3.3 The relationship between LBBAP pacing modes and location of the lead tip under fluoroscopy

**Figure 3:**
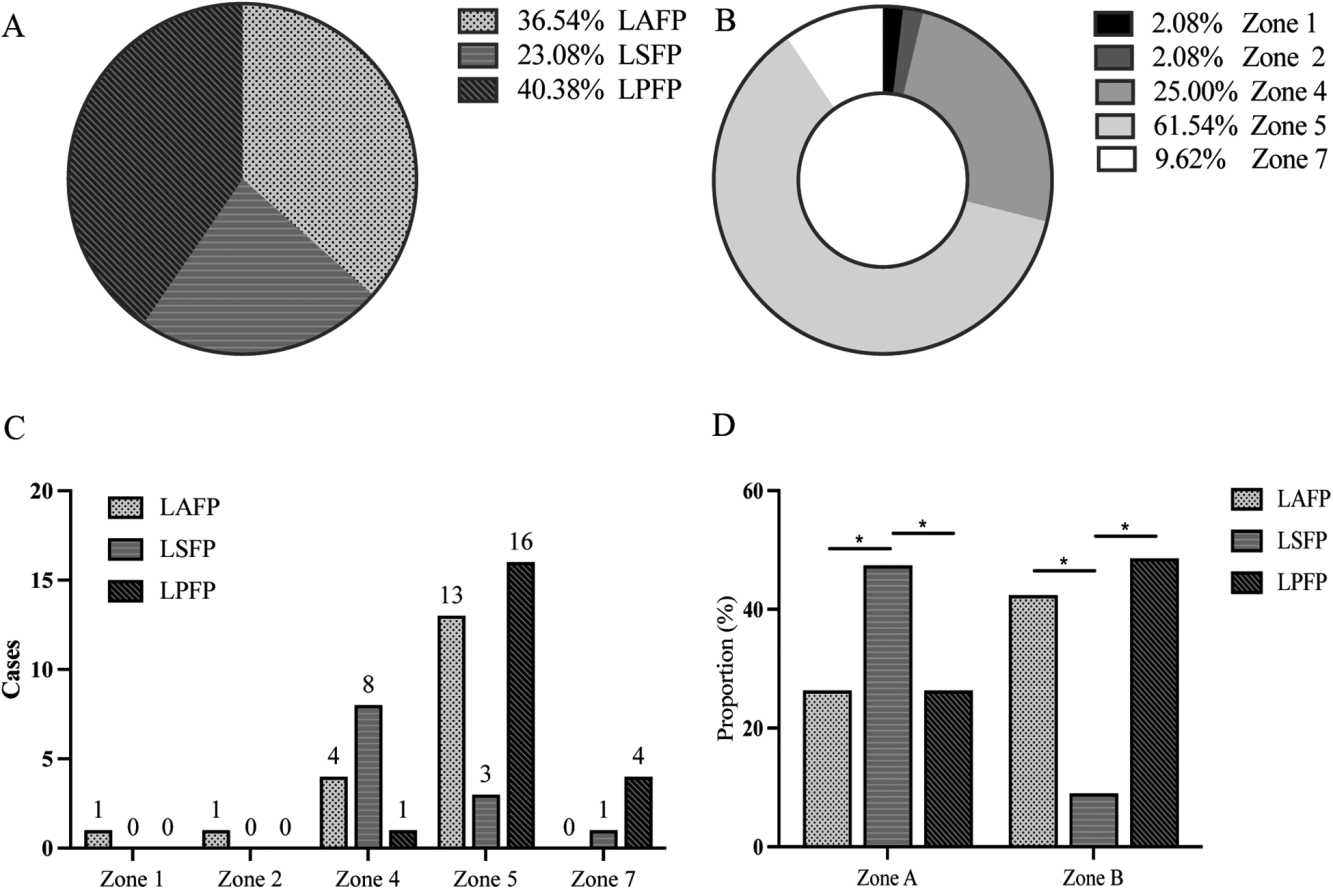
Correlation between different LBBAP modes and location of lead tip under fluoroscopy (*n* = 52). **(A)** Proportion of the three LBBAP modes among the study population; **(B)** Proportion of lead tip location among the study population; **(C)** The distribution of LBBAP modes in distinct zones; **(D)** Chi-square test of the correlation between LBBAP modes and the lead location. Section A consisted of Zone 1, Zone 4, and Zone 7; Section B was comprised of Zone 2 and Zone 5. LAFP, left anterior fascicle pacing; LBBAP, left bundle branch area pacing; LBTP, left bundle trunk pacing; LPFP, left posterior fascicle pacing. *P < 0.05.

Since 12 patients were excluded due to randomly missing image data, a total of 52 patients were finally included for further analysis in this section, with 19 in the LAFP group (36.5%), 12 in the LBTP group (23.1%), and 21 in the LPFP group (40.4%) (Figure 3A), and the proportion showed no statistical difference compared to the excluded ones (*P *= 0.61). Among the 52 patients, lead tips were most commonly located in Zone 5 (61.5%), followed by Zone 4 (25.0%), with the remaining 13.5% situated at Zone 1, 2, and 7. However, no lead tips were observed in Zone 3, 6, 8, and 9 (Figure 3B). There was a certain trend concerning the lead tip distribution in the two most commonly located zones: despite partial overlap among the three LBBAP groups, LAFP and LPFP were mostly distributed in Zone 5 while LBTP tended to locate in Zone 4. Interestingly, LAFP was the only pacing mode in Zone 1 and 2 (the high interventricular septum), while showed no presence in Zone 7 (the low interventricular septum) (Figure 3C).

In order to assess the statistical significance for the above distribution trend, the study further merged Zone 1, 4, and 7 into Section A, while the other two zones (2 and 5) into Section B based on their distance from the atrioventricular junction due to the small sample size. Chi-square test was then used to compare the distribution of three LBBAP modes in Sections A and B. The results showed a significant correlation (*P *= 0.01) between the pacing modes of LBBAP and the location of the lead tip: LBTP was more likely to be in Section A (LBTP vs. LASP: 47.4 vs. 26.3%; LBTP vs. LPFP: 47.4 vs. 26.3%; *P *< 0.05), while LPFP or LAFP tended to be located in Section B (LBTP vs. LASP: 9.1 vs. 42.4%; LBTP vs. LPFP: 9.1 vs. 48.5%; *P *< 0.05) (Figure 3D).

### 3.4 Influence of distinct LBBAP modes on ventricular depolarization and repolarization parameters

**Figure 4:**
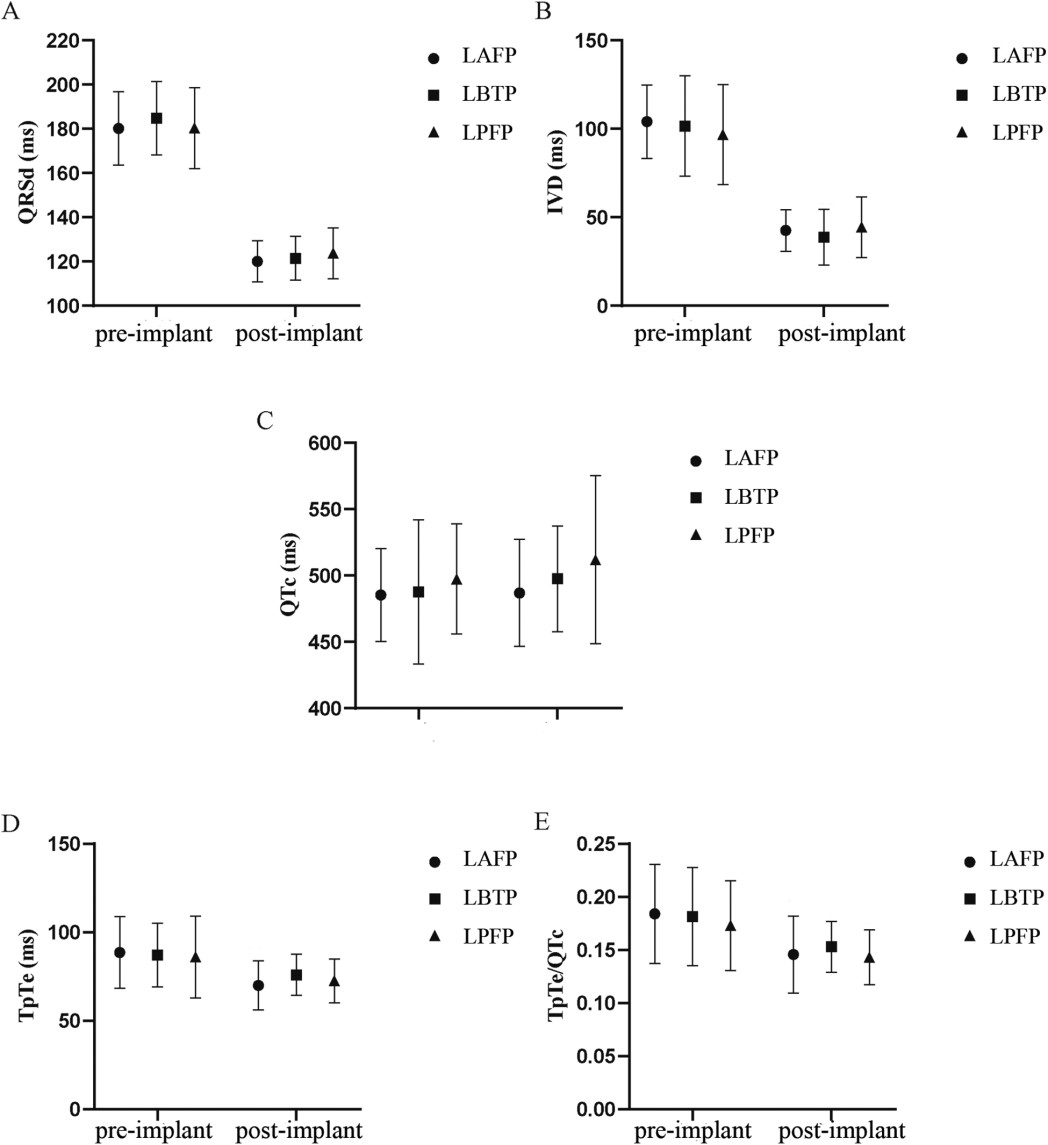
Comparison of QRSd **(A)**, IVD **(B)**, QTc **(C)**, TpTe (**D**), and TpTe/QTc **(E)** among different LBBAP modes (*n* = 64). IVD, interventricular delay; LAFP, left anterior fascicle pacing; LBBAP, left bundle branch area pacing; LBTP, left bundle trunk pacing; LPFP, left posterior fascicle pacing; QRSd, QRS duration; TpTe, TpeakTend.

LAFP, LBTP and LPFP all significantly improved ventricular depolarization synchrony and repolarization stability in HF patients: QRSd and IVD, the typical indicators of ventricular depolarization synchrony, were significantly shortened after LBBAP; The repolarization parameters TpTe and TpTe/QTc showed a significant decrease compared to baseline (except that TpTe after LBTP showed a trend with borderline significance), while no significant difference in QTc was observed (Table 3). Besides, there was no difference in both baseline and paced parameters among LAFP, LBTP and LPFP groups. In other words, the effect of distinct LBBAP modes on ventricular depolarization and repolarization parameters were similar between groups (Figure 4).

**Table 3:** Changes in ventricular depolarization and repolarization parameters before and after distinct LBBAP modes.

ECG parameters	Groups	Baseline	LBBAP	*P* value
QRSd (ms)	LAFP	184.87 ± 16.59	120.04 ± 9.28	<0.001***
	LBTP	184.87 ± 16.59	121.48 ± 9.91	<0.001***
	LPFP	180.39 ± 18.31	123.71 ± 11.49	<0.001***
IVD (ms)	LAFP	106.00 (90.00, 116.00)	41.00 (35.50, 52.00)	<0.001***
	LBTP	108.00 (92.00, 112.00)	36.00 (27.00, 51.50)	0.001**
	LPFP	100.00 (90.00, 117.00)	46.00 (35.50, 58.00)	<0.001***
TpTe (ms)	LAFP	85.00 (74.00, 99.10)	65.00 (58.00, 80.90)	<0.001***
	LBTP	87.00 (74.50, 97.10)	75.90 (68.00, 81.50)	0.058
	LPFP	82.00 (71.00, 92.50)	71.00 (63.50, 81.00)	0.005**
QTc(ms)	LAFP	485.39 ± 34.98	486.93 ± 40.35	0.86
	LBTP	487.64 ± 54.44	497.53 ± 39.72	0.52
	LPFP	497.44 ± 41.46	511.97 ± 63.22	0.22
TpTe/QTc	LAFP	0.17 (0.15, 0.22)	0.13 (0.11, 0.17)	0.001**
	LBTP	0.18 (0.14, 0.22)	0.15 (0.14, 0.16)	0.017*
	LPFP	0.16 (0.14, 0.19)	0.14 (0.12, 0.16)	0.001**

IVD, interventricular delay; LAFP, left anterior fascicle pacing; LBBAP, left bundle branch area pacing; LBTP, left bundle trunk pacing; LPFP, left posterior fascicle pacing; QRSd, QRS duration; TpTe, TpeakTend.

* *P *< 0.05.

** *P *< 0.01.

*** *P *< 0.001.

### 3.5 The correlation between distinct LBBAP modes and echocardiographic response

Only 52 patients were included in the analysis due to missing follow-up echocardiographic data in the remaining 12 patients. After a follow-up of 5.0 (3.0, 9.0) months, the overall response rate was 82.7% (43/52), and the super-response rate was 36.5% (19/52). LVEF was significantly increased (LVEF 47.15 ± 10.75 vs. 32.81 ± 6.79%, *P *< 0.001), and LVEDD was significantly reduced (LVEDD 56.44 ± 8.44 vs. 65.00 ± 8.94 mm, *P *< 0.001) after LBBAP. The improvement in echocardiographic data was more significant in responders than non-responders (Table 4).

**Table 4:** Comparison of echocardiographic data between responders and non-responders (*n* = 52).

Parameters	Responders (*n* = 43)	Non-responders (*n* = 9)	*P* value
Baseline LVEF (%)	32.14 ± 6.66	36.0 ± 6.86	0.12
Baseline LVEDD (mm)	64.74 ± 8.47	66.22 ± 11.45	0.66
Follow-up LVEF (%)	52.00 (40.00, 56.00)	40.00 (29.00, 46.00)	0.003**
Follow-up LVEDD (mm)	55.00 (50.00, 60.00)	57.00 (55.50, 70.00)	0.023*
Delta LVEF (%)	17.0 (8.00, 23.00)	2.0 (0.00, 4.00)	<0.001***
Delta LVEDD (mm)	9.0 (3.00, 13.00)	1.0(−2.50, 6.50)	0.03*

LVEDD, left ventricular end diastolic volume; LVEF, left ventricular ejection fraction; Delta LVEF, follow-up LVEF—baseline LVEF; Delta LVEDD, baseline LVEDD—follow-up LVEDD.

* *P *< 0.05.

** *P *< 0.01.

*** *P *< 0.001.

**Figure 5:**
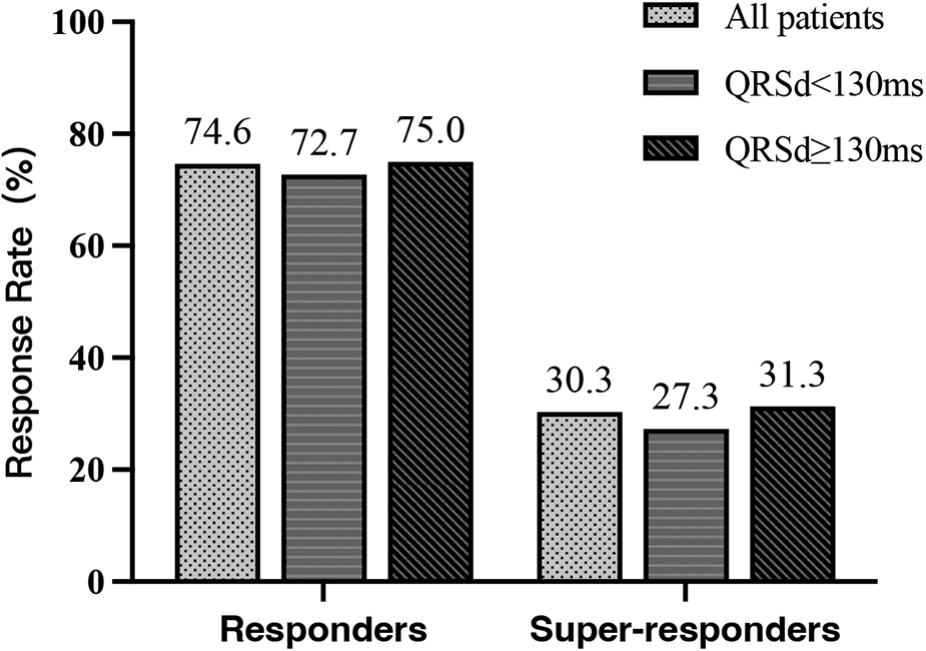
Comparison of echocardiographic response among LBTP group, LAFP group, and LPFP group (*n* = 52). LAFP, left anterior fascicle pacing; LBTP, left bundle trunk pacing; LPFP, left posterior fascicle pacing.

Among the 52 patients, a total of 13 (25.0%) had LBTP, 19 (36.5%) had LAFP, and 20 (38.5%) had LPFP. Baseline characteristics among the three groups were comparable, and there was no difference in echocardiographic response and super-response rate among groups (Table 5, Figure 5).

**Table 5:** Comparison of clinical and echocardiographic characteristics among LBTP group, LAFP group, and LPFP group (*n* = 52).

Variables	LBTP (*n* = 13)	LAFP (*n* = 19)	LPFP (*n* = 20)	*P* value
Clinical characteristics				
Gender (%, male)	8 (61.5)	11 (57.9)	14 (70.0)	0.76
Age (year)	60.00 (47.00, 68.00)	63.00 (57.00, 71.00)	58.50 (54.50, 67.50)	0.41
Hypertension (%)	5 (38.5)	10 (52.6)	10 (50.0)	0.78
Diabetes (%)	2 (10.5)	2 (15.4)	4 (20.0)	0.89
Af	2 (15.4)	3 (15.8)	1 (5.0)	0.55
CAD (%)	5 (38.5)	5 (26.3)	8 (40.0)	0.71
ICM(%)	2 (15.4)	4 (21.1)	2 (10.0)	0.72
Hyperlipidemia (%)	5 (38.5)	10 (52.6)	13 (65.0)	0.34
CKD (%)	0 (0.0)	3 (15.8)	1 (5.0)	0.35
LBBB (%)	9 (69.2)	13 (68.4)	15 (75.0)	0.93
Baseline QRSd	181.22 ± 14.61	178.42 ± 17.08	180.70 ± 20.03	0.89
Paced QRSd	120.36 ± 10.21	119.52 ± 9.87	120.52 ± 11.07	0.95
*β*-receptor bloker (%)	12 (92.3)	19 (100.0)	20 (100.0)	0.25
ACEI/ARB	11 (84.6)	14 (73.7)	18 (90.0)	0.42
Amiodarone (%)	6 (46.2)	5 (26.3)	4 (20.0)	0.29
Echocardiographic data				
Baseline LVEF (%)	31.92 ± 7.95	32.63 ± 5.74	33.55 ± 7.17	0.80
Baseline LVEDD (mm)	64.00 ± 5.82	64.68 ± 8.11	65.95 ± 11.37	0.82
Follow-up LVEF(%)	45.08 ± 11.41	49.21 ± 10.96	46.55 ± 10.33	0.55
Follow-up LVEDD (mm)	54.00 ± 10.05	56.95 ± 8.21	57.55 ± 7.61	0.48
Delta LVEF (%)	10.00 (7.00, 22.00)	13.00 (7.00, 27.00)	8.5 (4.25, 20.50)	0.31
DeltaLVEDD (mm)	9.00 (3.50, 13.50)	8.00 (4.00, 12.00)	5.50 (4.25, 20.50)	0.73
Responders (%)	11 (84.6)	17 (89.5)	15 (75.0)	0.53
Super-responders (%)	5 (38.5)	8 (42.1)	6 (30.0)	0.76
**Follow-up time(month)**	6.58 ± 3.76	8.08 ± 4.77	6.05 ± 3.99	0.40
**Pacing percentage (%)**	99.90 (99.20, 100.00)	99.90 (98.30, 100.00)	99.40 (97.70, 100.00)	0.51

ACEI/ARB, angiotensin converting enzyme inhibitor/angiotensin receptor antagonist; Af, atrial fibrillation; CAD, coronary artery disease; CKD, chronic kidney disease; ICM, ischemic cardiomyopathy; LAFP, left anterior branch pacing; LBTP, left bundle branch main pacing; LBBB, left bundle branch block; LPFP, left posterior branch pacing; LVEDD, left ventricular end diastolic diameter; LVEF, left ventricular ejection fraction; QRSd, QRS duration; Delta LVEF, follow-up LVEF—baseline LVEF; Delta LVEDD, baseline LVEDD—follow-up LVEDD.

## 4 Discussion

This study evaluated elaborately the effects of distinct LBBAP modes on ECG parameters and echocardiographic response among HF patients indicated for CRT. The main findings were as follows: (1) LPFP was the most common LBBAP mode in HF patients; (2) The tip of LBBAP lead was mainly distributed in Zone 4 and 5, and its location was relevant to different pacing modes; (3) Distinct LBBAP modes improved ventricular depolarization synchrony and repolarization stability equally in HF patients. (4) No significant difference was observed in echocardiographic response and super-response rate among different LBBAP modes.

### 4.1 Distribution of distinct LBBAP modes in HF patients

Due to the fan-shaped distribution of LBB underneath the LV intima, LBBAP could be achieved in a wide area of the interventricular septum during the procedure both theoretically and practically (2). Although this notion facilitated the novel technique, reduced septum injury resulting from repeated attempts, shortened radiation time, it also led to high heterogeneity of LBBAP. Various LBBAP modes such as LBTP, LAFP, and LPFP emerged (2). The proportion of these different LBBAP modes among patients with normal cardiac function has been reported in several studies, with LPFP being the most common mode. Zhang et al. (22) conducted electrophysiological mapping on LBBAP patients and found that most of the lead tips were located in the area of the left posterior branch. Lin et al. (15) reported that in 68 AVB patients undergoing successful LBBAP, LPFP accounted for 51.5% of the total population, LBTP and LAFP accounted for 25 and 23.5% respectively. The predominance of LPFP in these studies may be due to the fact that LPF is the main continuation of LBB with fan-shaped distribution in a wide area of low-to-medium septum under LV intima, making it easier to be targeted (12, 15). Things may be different in HF patients due to the development of electrical-mechanical remodeling. This study was the first to evaluate the distribution of distinct pacing modes in HF patients, and it was found that LPFP was also the pacing mode with the highest proportion (40.6%), consistent with the previous study involving patients with normal cardiac function.

### 4.2 Distribution of the LBBAP lead Tip under fluoroscopy in HF patients

Researchers have studied the distribution of LBBAP lead tip in the interventricular septum under fluoroscopy among successful cases to further refine operation techniques and improve success rate (13, 22). For patients with normal cardiac function, the lead tip of LBBAP is mainly distributed in the 4th and 5th zones according to the nine-partition method (15, 22). Studies have reported that HF (OR: 1.49, 95% CI: 1.01–2.21, *P* < 0.05) and LVEDD (OR: 1.53, 95% CI: 1.26–1.86, *P* < 0.001) are independent predictive factors for failed LBBAP implantation (2). Due to cardiac electrical and mechanical remodeling, the operation techniques of LBBAP among HF patients may differ from those with normal cardiac function, and whether the aforementioned nine-partition method still works for HF patients remains to be further clarified. This study firstly evaluated the distribution of lead tips in HF patients and found that most tips also distributed in zones 4 and 5, which may be due to the fact that nine-zone method indicated relative rather than absolute position of tips on the interventricular septum.

Furthermore, this study found that anatomical zones were significantly correlated with pacing patterns, with zone 4 or A zone (mainly including zone 4) more likely to have LBTP, while zone 5 or B zone (mainly including zone 5) more likely to exhibit LPFP and LAFP. The main difference in the distribution of zone 4 and 5 on the interventricular septum lies in the distance from the atrioventricular junction, with zone 4 located in the proximal 1/3 and zone 5 located in the middle 1/3. This correlation between anatomical zones and pacing patterns may be explained by the anatomical characteristics of LBB, namely that the LBB trunk goes approximately 10–15 mm and divides into LAF and LPF afterwards (12). Consistently, Liu et al. (16) found that in the fan-shaped area 1(similar to the 4th zone in our study), located 15–35 mm from the tricuspid valve (TV), LBFP was the main pattern; while in the area 2 (basically comprised of the zone 5 and 8 in our study), located 35–50 mm from TV, branch pacing was more common.

In addition, this study found that zone 1 and zone 2 (the upper interventricular septum) had only LAFP while zone 7 (the lower interventricular septum) contained the other two pacing patterns without LAFP, corresponding to the anatomical distribution of LBB branches. LAF goes across LV outflow tract upwards and forwards and terminates at the base of the anterior papillary muscle after emerging from the LBB trunk, while the LPF runs backwards and downwards to the base of the posterior papillary muscle (12). Overall, location of the LBBAP lead tip under fluoroscopy on the RV interventricular septum could predict the final pacing pattern to a certain extent.

### 4.3 Effects of distinct LBBAP modes on ECG parameters and echocardiographic response of HF patients

Among patients with narrow QRS and normal cardiac function, studies have been carried out concerning comparison of depolarization synchrony between distinct LBBAP pacing modes, although with controversial results. Lin et al. (15) believe that LBTP, LAFP, and LPFP could achieve equivalent electrical synchrony, while Liu et al. (16) found that LBTP could obtain shorter QRSd and smaller QRS area than branch pacing. Several studies involving HF patients have reported that LBBAP improved depolarization synchrony (5–7, 31, 32), but whether the effects differ between distinct pacing modes has not been reported yet. Our study compared for the first time the ventricular depolarization parameters among distinct LBBAP modes in HF patients with wide QRS, the typical candidates for CRT. The results turned out that the QRSd and IVD of the three groups (LBTP, LPFP, and LAFP) significantly decreased after LBBAP without intergroup difference, suggesting equal improvement of the electrical synchrony. This may be related to the numerous ramus communicans of the LBB conduction system, resulting in rapid capture of the entire conduction system (12, 15). In addition, this study further compared the improvement of cardiac function among distinct pacing modes and no significant difference was observed in the echocardiographic response and super-response among the three groups, consistent with the electrical synchrony.

The repolarization stability in HF patients is also of vital significance except for depolarization, since repolarization is supposed to be closely related to VAs/SCD. Most HF patients who meet the indications for CRT also meet the class I indication for implantable cardioverter defibrillator (ICD) (33). Besides, pacing itself can also affect repolarization stability and thus increase the risk of VAs/SCD. For example, the dispersion of ventricular repolarization (DVR) increased early after BiVP, meanwhile the risk of VAs also increased (25). However, there is currently limited evidence about the influence of different LBBAP pacing modes on DVR. Only one small study involving patients with narrow QRS and normal cardiac function found slight difference in the QT interval among various branch pacing modes (LBTP vs. LPFP vs. LAFP: 363.6 ± 40.4 vs. 392.9 ± 45.0 vs. 370.8 ± 32.5 ms, *P* = 0.037), while the QTc showed no difference (15). Our study found that the repolarization parameters QTc, TpTe, and TpTe/QTc of the three groups all significantly and equally decreased after pacing, indicating that distinct pacing modes may have an equally beneficial effect on the repolarization stability of HF patients with wide QRS. However, whether the risks of VAs/SCD among the three groups differ still needs further investigation.

In conclusion, this study found that in HF patients undergoing successful LBBAP, different pacing modes could achieve equally high depolarization synchronicity, repolarization stability, and echocardiographic response, with stable pacing parameters during follow-up. Therefore, it is unnecessary to seek to capture the LBB trunk or specific branches when performing LBBAP in HF patients, in order to shorten procedure time and reduce damage of the septum due to repeated attempts.

## 5 Limitations analysis

Firstly, this study is a retrospective study with relatively small sample size, and the results still need to be further validated by a larger sample study. Due to the short reassessment period and small subject numbers, the absence of significant differences between LBBAP pacing types and outcomes can be due to Type II errors. Secondly, this study only compared the three pacing modes from the aspects of electrical and echocardiographic response, whether the clinical response, ultimate survival and other outcomes of HF patients differ needs further research. Thirdly, the long-term changes in DVR, the possible difference in VAs/SCD events, and long-term response rates among LBBAP modes warrantee further investigation.

## 6 Conclusion

LPFP was the main pacing mode among HF patients after LBBAP. The paced QRS morphology was significantly related to the position of lead tips. After LBBAP, the ventricular depolarization synchronization and repolarization stability were both significantly improved, regardless of pacing modes. There was no significant difference in the echocardiographic response rate among distinct LBBAP modes during short-term follow-up.

## Data Availability

The raw data supporting the conclusions of this article will be made available by the authors, without undue reservation.
